# Supplementary material for: Characterization of Fibrodysplasia Ossificans Progessiva relevant Acvr1/Acvr2 Activin receptors in medaka (Oryzias latipes)
Source: PLoS One. 2023 Sep 14;18(9):e0291379. doi: 10.1371/journal.pone.0291379 (PMC10501582; doi:10.1371/journal.pone.0291379)
Supplement: S1 Table — (DOCX) [file pone.0291379.s001.docx]

Supplementary Table S1: Oligonucleotides used for cloning of *in situ* riboprobes

| **Name** | **Oligonucleotide-sequence (5'-3')** | |
| --- | --- | --- |
|  | **for** | **rev** |
| *olaAlk1* | ACCTTGCAGACTTTGCATCAAT | AACGCCAAATCATCACACCGA |
| *olaAcvr1* | AGATCATGTGCTTTCCGCCT | CATGACTGCAAGTCCGAGGT |
| *olaAcvr1l* | GTGAAGGCCCAAAATGCCTC | CTGGGTGCTGGAGTTACGAG |
| *olaAcvr2ab* | ATTTGTGTCTTGTTGCCCCG | AGTATGCTGGAAGCGTGACA |
| *olaAcvr2ba* | CACCAGCATCCAGGTGCTAA | TCTGAAAGTTGATGGCCCCC |
| *olaAcvr2bb* | TGTGCTTTGACCGTGGTGAT | CGTCCACCCCATAAGACGAT |
| *olaAlk1* | ACTGACCATCCCTCCACGTA | CGCATTGCTGGAACGTTGTT |
