# Supplementary material for: Characterization of Fibrodysplasia Ossificans Progessiva relevant Acvr1/Acvr2 Activin receptors in medaka (Oryzias latipes)
Source: PLoS One. 2023 Sep 14;18(9):e0291379. doi: 10.1371/journal.pone.0291379 (PMC10501582; doi:10.1371/journal.pone.0291379)
Supplement: S2 Table — (DOCX) [file pone.0291379.s002.docx]

Supplementary Table S2: Oligonucleotides used for cloning of Halo-tag plasmids

| **Name** | **Oligonucleotide-sequence (5'-3')** | |
| --- | --- | --- |
|  | **for** | **rev** |
| *olaAlk1* | GGTGGAGAATTCGACAGCAACGATGATGCAAATGACAGG | CCACCTGCGGCCGCGATGTCCTGCTTGACTTTGCTGAG |
| *olaAcvr1* | GGTGGAGAATTCTTGGACGGCATGAGCGGTTTGACAAACGCC | CCACCTGCGGCCGCAATGTCTGTTTTGATCTTATCC |
| *olaAcvr1l* | GGTGGAGAATTCGAAGGCTCAGATGTACATCTGG | CCACCTGCGGCCGCCGACTCCTTGCCCTTCTCAAGAGAGC |
| *olaAcvr2ab* | GGTGGAGAATTCCGCTCCGAGACTCAGAAGTGC | GCTCGAGCGGCCGCTCATAGACTAGACTCCTTTGG |
| *olaAcvr2ba* | GGTGGAGAATTCGGGCTGAGTGGAGGCGGGGC | GCTCGAGCGGCCGCTCAAATGCTGGACTCTTTGG |
| *olaAcvr2bb* | GGTGGAGAATTCGAAGTGGAAACGCGGGAGTGC | GCTCGAGCGGCCGCTCAGGAGCTGGACTCTTTGG |
