# Supplementary figures and images for: Characterization of Fibrodysplasia Ossificans Progessiva relevant Acvr1/Acvr2 Activin receptors in medaka (Oryzias latipes)
Source: PLoS One. 2023 Sep 14;18(9):e0291379. doi: 10.1371/journal.pone.0291379 (PMC10501582; doi:10.1371/journal.pone.0291379)

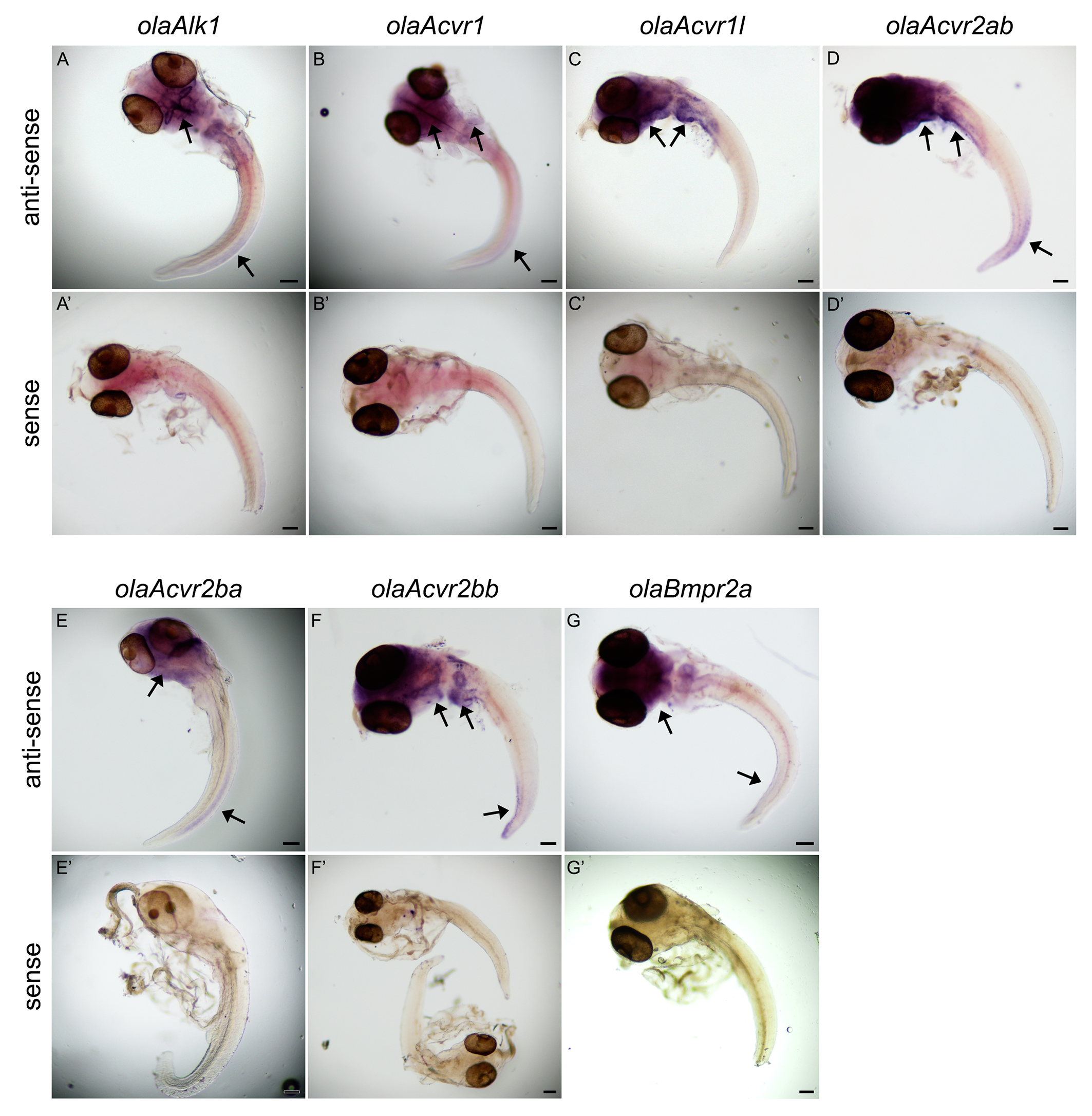

Supplement: S1 Fig — (A-G) Comparison of anti-sense riboprobe staining for olaAlk1, olaAcvr1, olaAcvr1l, olaAcvr2ab, olaAcvr2ba, olaAcvr2bb and olaBmpr2a with that of respective sense riboprobes (A’-G’) by whole mount RNA in situ hybridization of 5 dpf medaka embryos. Arrows mark stained structures. Scale bars = 100 μm. (TIF) [file pone.0291379.s003.tif]

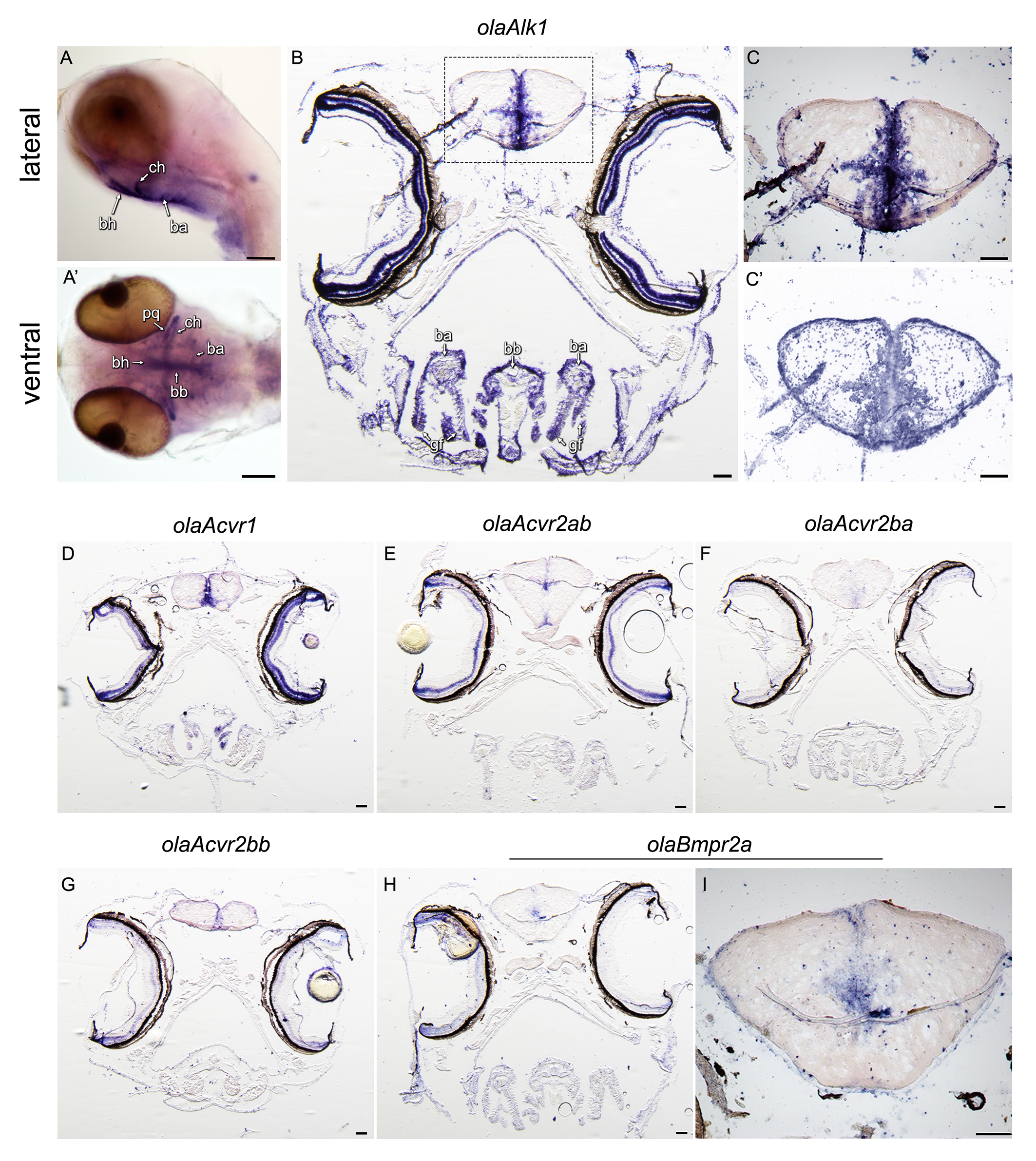

Supplement: S2 Fig — (A, A’) olaAlk1 expression pattern by whole mount RNA in situ hybridization in 5 dpf medaka embryos. (B) Overview of olaAlk1 expression pattern in 2 mpf medaka head by RNA in situ hybridization on cryosections. (C, C’) Magnified views of box marked in B, showing (C) olaAlk1 expression and (C’) DAPI staining (pseudo-colored). (D-H) Comparison of olaAcvr1, olaAcvr2ab, olaAcvr2ba, olaAcvr2bb and olaBmpr2a expression in 2 mpf medaka head sections by RNA in situ hybridization. (I) olaBmpr2a expression pattern in 2 mpf medaka brain by RNA in situ hybridization on cryosections. ba—branchial arch, bb–basibranchial, bh–basihyal, ch–ceratohyal, gf—gill filaments, pq–palatoquadrate. Scale bars = 100 μm. (TIF) [file pone.0291379.s004.tif]

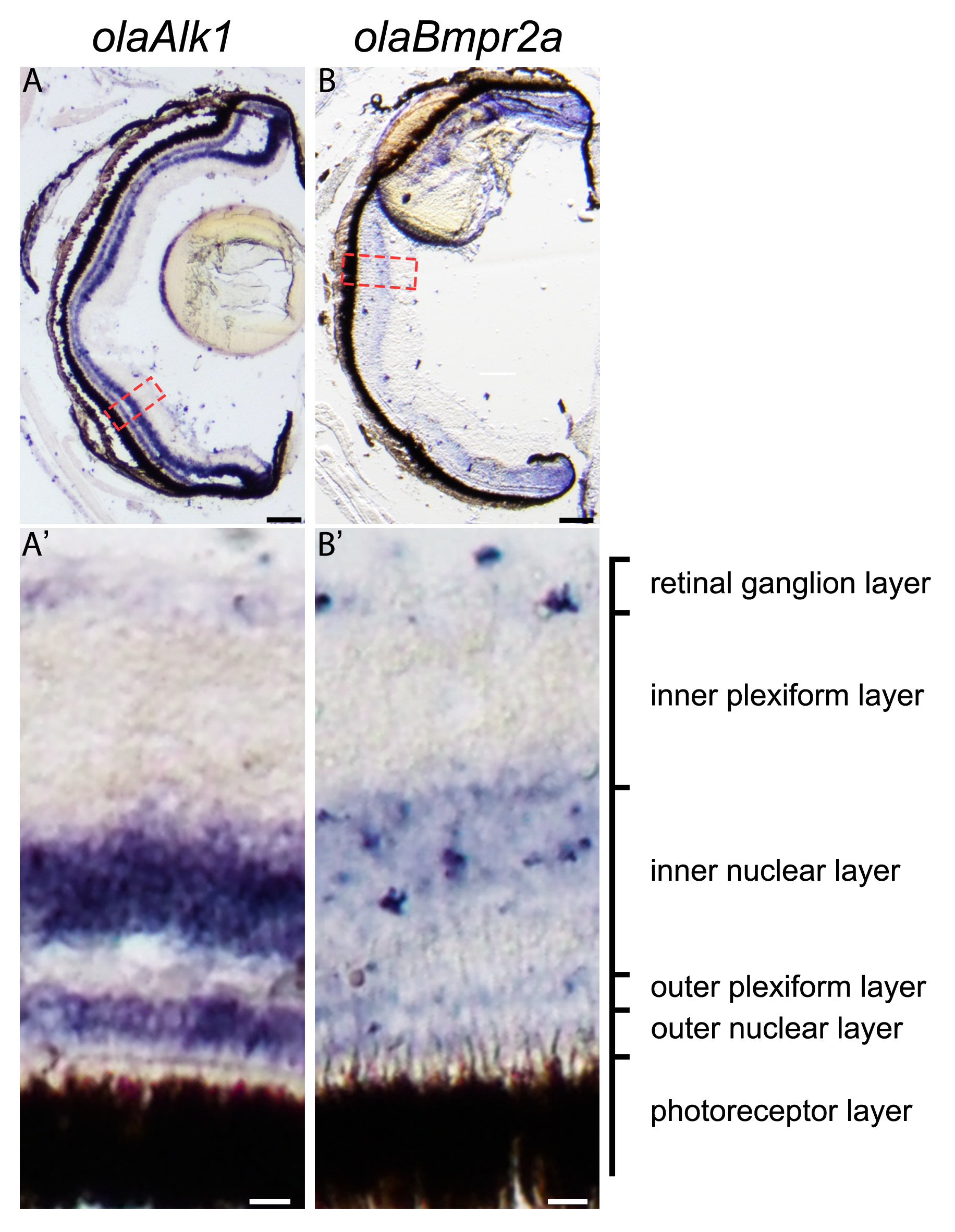

Supplement: S3 Fig — Comparison of olaAlk1 and olaBmpr2a expression in 2 mpf medaka eyes by RNA in situ hybridization on cryosections. (A, B) Overviews of respective receptor mRNA staining. (A’-B’) Zoom in on area marked by red boxes in (A, B). Scale bars (A-B) = 100 μm, (A’-B’) = 10 μm. (TIF) [file pone.0291379.s005.tif]

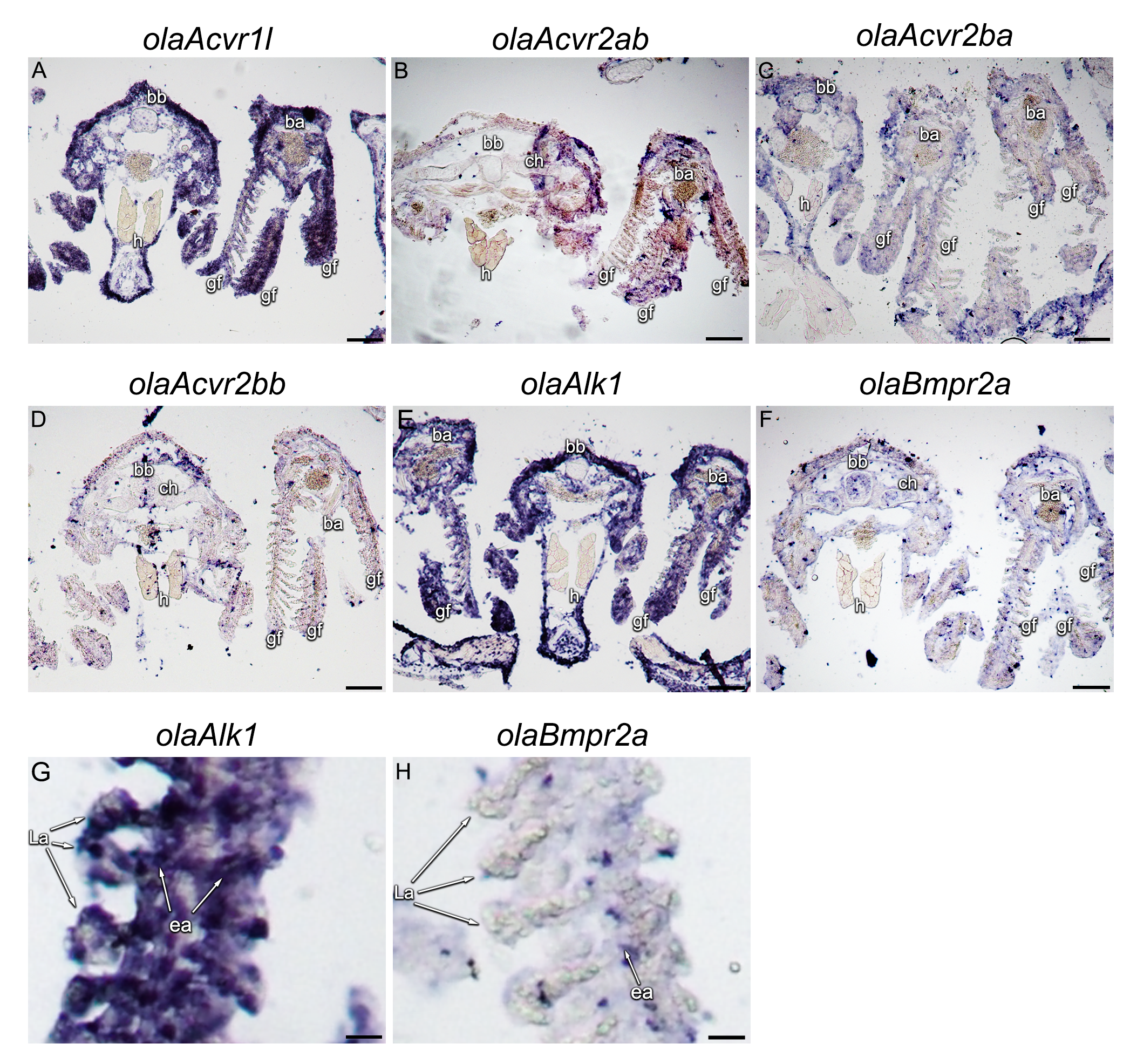

Supplement: S4 Fig — (A-F) Overview of olaAcvr1l, olaAcvr2ab, olaAcvr2ba, olaAcvr2bb, olaAlk1 and olaBmpr2a expression by RNA in situ hybridization on cryosections. (G-H) Zoom-in view of olaAlk1 and olaBmpr2a expression in 2 mpf medaka gills. ba–branchial arch, bb–basibranchial, bh–basihyal, ch–ceratohyal, ea–efferent artery, gf–gill filaments, h–heart, L–lamellae. Scale bars (A-F) = 100 μm, (G, H) = 15 μm. (TIF) [file pone.0291379.s006.tif]

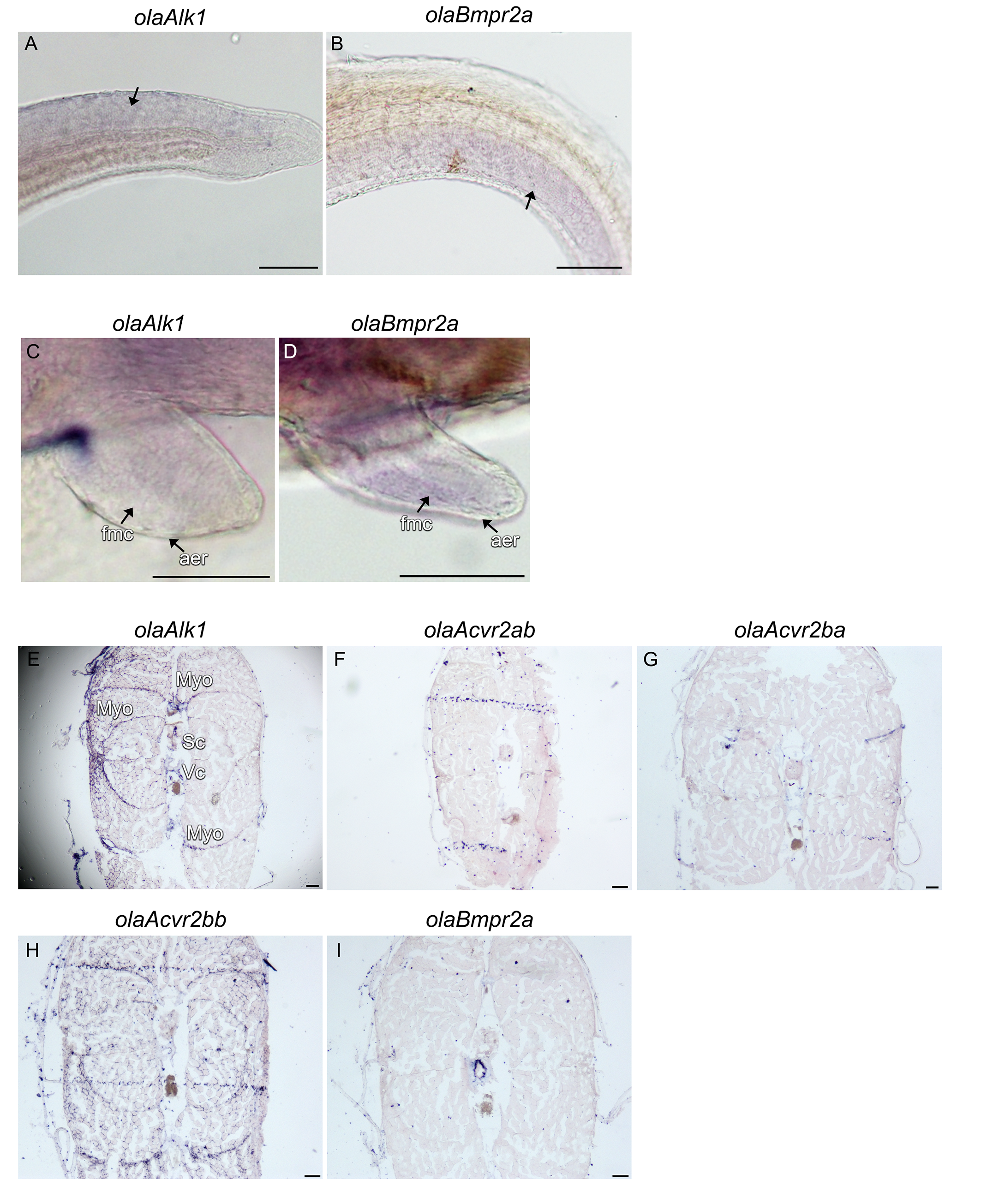

Supplement: S5 Fig — (A—D) Comparison of olaAlk1 and olaBmpr2a expression by whole mount RNA in situ hybridization in 5 dpf medaka trunk and pectoral fin tissue. (E-H) Comparison of olaAlk1, olaAcvr2ab, olaAcvr2ba, olaAcvr2bb and olaBmpr2a expression in cryosections of 2 mpf medaka trunks by RNA in situ hybridization. aer–apical ectodermal ridge, fmc–fin mesenchyme, Myo–myosepta, Sc–spinal cord, Vc–vertebral column. Scale bars = 100 μm. (TIF) [file pone.0291379.s007.tif]

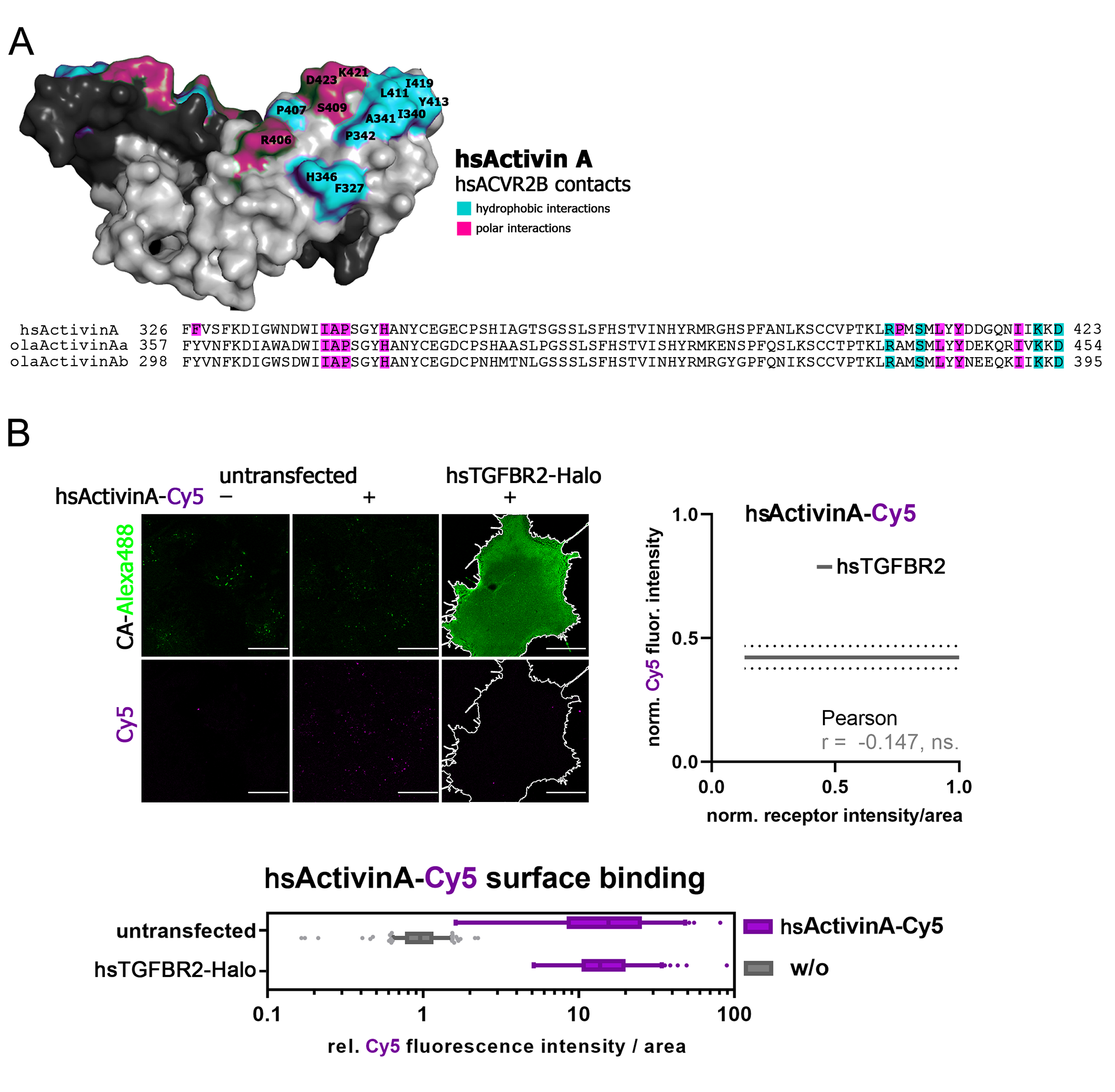

Supplement: S6 Fig — (A) Alignment of hsActivin A ligand binding domain sequence with that of respective medaka ligands; polar receptor:ligand interaction sites are in light magenta and hydrophobic interaction sites in teal (PDB 1S4Y). (B) For negative control, transiently transfected COS-7 cells expressing Halo-tagged hsTGFBR2 receptors were simultaneously incubated with Halo-tag substrate CA-Alexa488 (green) and hsActivin A-Cy5 (magenta) at 24 hours post transfection (n = 3 independent experiments). (B upper left) Representative confocal microscopy images of COS-7 cells transiently expressing respective receptors, as well as untransfected controls. (B bottom) hsActivin A-Cy5 surface binding represented as relative fluorescence intensity per area to untransfected unstimulated control. Data is shown as fold induction ± standard deviation. Significance was calculated using two-way ANOVA and Tukey’s post-hoc test. ***p < 0.001 ***, p <0.0001 ≡ significance as indicated (n = 3). (B upper right) Linear regression and correlation analysis of ligand:receptor binding based on Cy5-fluorescence intensity and normalized receptor fluorescence (CA-Alexa488) per area (n = 3). Correlation was analyzed using the Pearson Test (p < 0.0001 ≡ ****, p < 0.001 ≡ ***, p < 0.01 ≡ **, p < 0.5 ≡ *, p > 0.5 ≡ ns.). Scale bar = 20 μm. (TIF) [file pone.0291379.s008.tif]

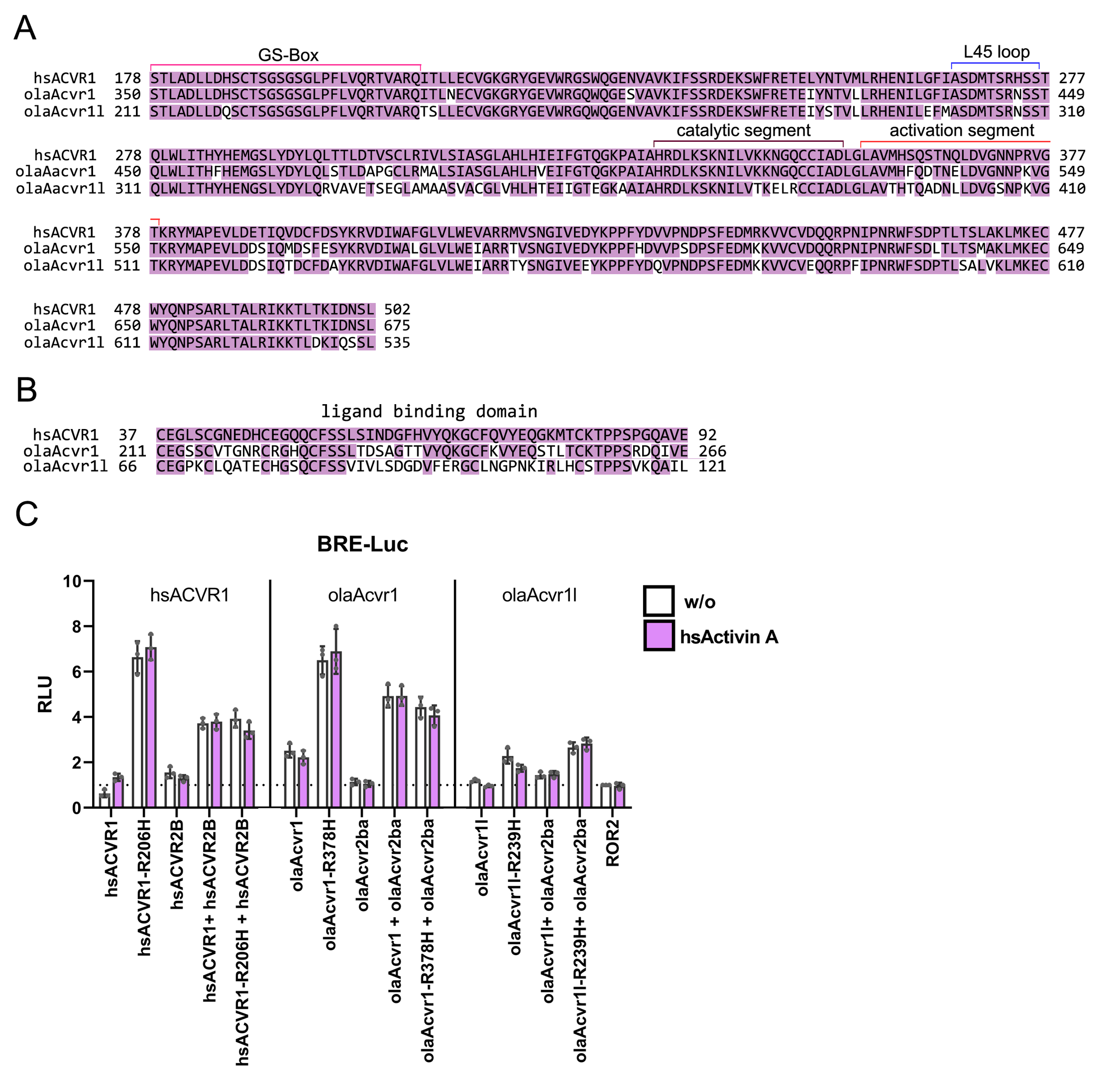

Supplement: S7 Fig — (A) Alignment of hsACVR1 ligand binding domain sequence with that of respective medaka receptors; similarities are highlighted in magenta. (B) Alignment of entire hsACVR1 kinase domain with that of respective medaka receptors; similarities are highlighted in magenta. (C) Luciferase activity one day after transfection of a SMAD1/5/8-sensitive BRE2-luciferase reporter and hRluc/TK together with either individual Halo-tagged hsACVR1, hsACVR1-R206H, olaAcvr1, olaAcvr1-R378H, olaAcvr1l, olaAcvr1l-R239H, or after co-transfection with hsACVR2B or olaAcvr2ba, respectively. ROR2 was used as control for endogenous signaling. After starvation for 3 h, HEK293T cells were stimulated overnight with hsActivin A (5 nM). Relative Luminescence Units (RLU) are expressed as mean fold induction ± standard deviation (n = 3 independent experiments). Statistical significance relative to no-stimulation control (w/o) was calculated using two-way ANOVA and Šidák multiple comparison test post-hoc test. (TIF) [file pone.0291379.s009.tif]

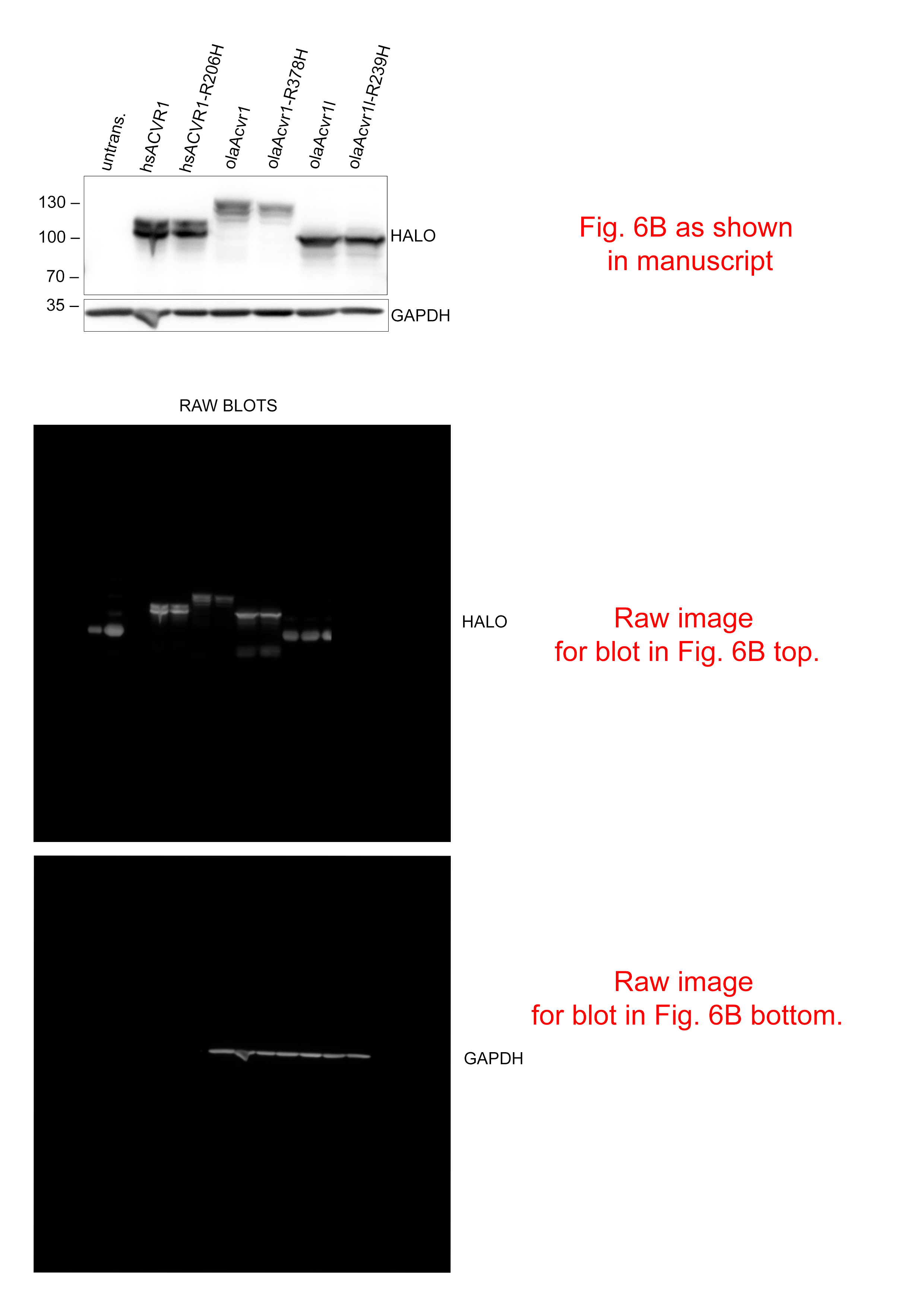

Supplement: S1 Raw images — (TIF) [file pone.0291379.s010.tif]
