# Supplementary material for: Characterization of Fibrodysplasia Ossificans Progessiva relevant Acvr1/Acvr2 Activin receptors in medaka (Oryzias latipes)
Source: PLoS One. 2023 Sep 14;18(9):e0291379. doi: 10.1371/journal.pone.0291379 (PMC10501582; doi:10.1371/journal.pone.0291379)
Supplement: Attachment — Submitted filename: PONE-D-22-34209_review.pdf [file pone.0291379.s011.pdf]

The manuscript provides an in-depth characterization of the two activin receptors in the small teleost model *Oryzias latipes*. The expression and the functional implication are clearly stated and the results support the conclusions. My major concern is about promoting an animal model for fibrodysplasia ossificans progressiva (FOP), which is not strictly the scope of this paper. First of all, I would suggest changing the title, because is misleading. The authors have not yet developed an animal model and no studies on medaka bone are reported in the paper. Thus, although it is clear that the study represents the 'background work' for establishing an animal model, it should be clearly specified (already in the title) that the work is only about the characterization of specific genes in a model species. As mentioned in the last paragraph of the abstract, it is preliminary work necessary before generating an animal model. This should be kept in mind throughout the manuscript. Thus, the scope of the introduction and the discussion should be reframed accordingly.

In addition, the following points should be addressed in the reviewed manuscript:

Line 59: TGFβ '-' missing (TGF-β)

The relationship between type I Activin receptors and SMAD activation is unclear and sometimes controversial in the introduction. Example: Lines 72-73 seem in contrast with lines 67-68. Previously ACVR1 was referred to activate SMAD1/5/8 (line 68), later as 'unable to transmit SMAD signals (73)'.

The authors should consider changing the order of some words to make rearing easier and more fluent. Example: - line 82: 'to study the mutated FOP causing ACVR1 receptor, an...' change to 'to study the ACVR1 receptor causing FOP...'. Check throughout the manuscript.

Avoid very long sentences (i.e. lines 208-213).

Line 93. Specify 'other skeletal phenotypes' in max 1 sentence.

In the introduction, especially when describing the previous zebrafish models, it is mentioned that important insights into the disease were recapitulated, but no skeletal phenotype is described. The focus is rather set on molecular pathways. Please add 1-2 lines on what could be mimicked and what not to highlight the need for a new animal model.

Line 123: the acronym olaAlk1 is never explained in the manuscript. It is only mentioned that it is used as a control.

Because most of the paper focuses on the expression pattern of medaka activin receptors type I and II, the introduction should contain some background info on what is known about the expression of the orthologues, at least in zebrafish, possibly in mouse. Other sections can be shortened (i.e. the section on invertebrates/D. melanogaster).

Results, I: The expression of olaAlk1 is detected in brain, branchial arches, blood vessels, ... 'close to skeletal structures'. Never observed expressed by skeletal structures in the head. How do you explain that? Maybe due to the observation timepoints?

Results, II: no expression of olaAcvr1/2 was detected in the gills at 5 days post-fertilization? If so, specify.

line 189: specify what fins

Concerning the experiments in cells, it would have been more consistent to use a medaka soft tissue cell line instead of a monkey line.

Line 493 should be euthanasia, not anesthesia, I hope.

For in situ hybridization on non-decalcified sections, shouldn't the samples be treated with levamisole to avoid detection of endogenous ALP activity (i.e. in bone)?
