# Supplementary material for: Characterization of Fibrodysplasia Ossificans Progessiva relevant Acvr1/Acvr2 Activin receptors in medaka (Oryzias latipes)
Source: PLoS One. 2023 Sep 14;18(9):e0291379. doi: 10.1371/journal.pone.0291379 (PMC10501582; doi:10.1371/journal.pone.0291379)
Supplement: Attachment — Submitted filename: Response to reviewer.docx [file pone.0291379.s013.docx]

Response to reviewers:

As requested, we have included a detailed protocol for the used whole mount RNA in-situ hybridization.
